# Symptoms of burnout in intensive care unit specialists facing the COVID-19 outbreak

**DOI:** 10.1186/s13613-020-00722-3

**Published:** 2020-08-08

**Authors:** Elie Azoulay, Jan De Waele, Ricard Ferrer, Thomas Staudinger, Marta Borkowska, Pedro Povoa, Katerina Iliopoulou, Antonio Artigas, Stefan J. Schaller, Manu Shankar Hari, Mariangela Pellegrini, Michael Darmon, Jozef Kesecioglu, Maurizio Cecconi, ESICM

**Affiliations:** 1 Médecine Intensive et Réanimation, PHP, Hôpital Saint-Louis, Paris University, Paris, France; 2 Department of Critical Care Medicine, Ghent University Hospital, 9000 Gent, The Netherlands; 3 Shock, Organ Dysfunction, and Resuscitation Research Group (SODIR), Instituto de Investigación de Vall d’Hebron, Barcelona, Spain; 4 Departmento de Medicina Intensiva, Hospital Universitario de Vall d́Hebron, Centro de Investigación Biomédica en Red (CIBER) de Enfermedades Respiratorias, Barcelona, Spain; 5 Department of Medicine I, Intensive Care Unit, Medical University of Vienna, Vienna General Hospital, Vienna, Austria; 6 NOVA Medical School, CHRC, New University of Lisbon, Lisbon, Portugal; 7 Unidade de Cuidados Intensivos Polivalente, Hospital de São Francisco Xavier, CHLO, Estrada Do Forte Do Alto Do Duque, 1449-005 Lisbon, Portugal; 8 Hellenic Army, ICU Nurse Manager General Military Hospital, Athens, Greece; 9 Critical Care Center, Sabadell Hospital, University Institute Parc Taulí, Autonomous University of Barcelona, Ciberes, Barcelona, Spain; 10 Department of Anesthesiology and Intensive Care, School of Medicine, Klinikum rechts der Isar, Technical University of Munich, Munich, Germany; 11 School of Immunology and Microbial Science, Kings College London, London, UK; 12 Guy’s and St Thomas’ NHS Foundation Trust, ICU Support Offices, St Thomas’ Hospital, London, UK; 13 Department of Surgical Sciences and Central Intensive Care Unit, Department of Anesthesia, Operation, and Intensive Care and Department of Anesthesiology and Intensive Care Medicine, Institute of Clinical Sciences, Sahlgrenska Academy, University of Gothenburg, Gothenburg, Sweden; 14 Department of Intensive Care Medicine, Division of Anesthesiology, Intensive Care and Emergency Medicine, University Medical Center Utrecht, Utrecht University, Utrecht, The Netherlands; 15 Humanitas Clinical and Research Center, Humanitas University, Milan, Italy

**Keywords:** Coronavirus, Pneumonia, Acute respiratory distress syndrome, Exhaustion, Depersonalization, Well-being

## Abstract

**Background:**

The COVID-19 pandemic has resulted in an unprecedented healthcare crisis with a high prevalence of psychological distress in healthcare providers. We sought to document the prevalence of burnout syndrome amongst intensivists facing the COVID-19 outbreak.

**Methods:**

Cross-sectional survey among intensivists part of the European Society of Intensive Care Medicine. Symptoms of severe burnout, anxiety and depression were collected. Factors independently associated with severe burnout were assessed using Cox model.

**Results:**

Response rate was 20% (1001 completed questionnaires were returned, 45 years [39–53], 34% women, from 85 countries, 12 regions, 50% university-affiliated hospitals). The prevalence of symptoms of anxiety and depression or severe burnout was 46.5%, 30.2%, and 51%, respectively, and varied significantly across regions. Rating of the relationship between intensivists and other ICU stakeholders differed significantly according to the presence of anxiety, depression, or burnout. Similar figures were reported for their rating of the ethical climate or the quality of the decision-making. Factors independently associated with anxiety were female gender (HR 1.85 [1.33–2.55]), working in a university-affiliated hospital (HR 0.58 [0.42–0.80]), living in a city of > 1 million inhabitants (HR 1.40 [1.01–1.94]), and clinician’s rating of the ethical climate (HR 0.83 [0.77–0.90]). Independent determinants of depression included female gender (HR 1.63 [1.15–2.31]) and clinician’s rating of the ethical climate (HR 0.84 [0.78–0.92]). Factors independently associated with symptoms of severe burnout included age (HR 0.98/year [0.97–0.99]) and clinician’s rating of the ethical climate (HR 0.76 [0.69–0.82]).

**Conclusions:**

The COVID-19 pandemic has had an overwhelming psychological impact on intensivists. Follow-up, and management are warranted to assess long-term psychological outcomes and alleviate the psychological burden of the pandemic on frontline personnel.

## Introduction

With more than six million confirmed cases worldwide and more than 350,000 deaths between February and May 2020, the COVID-19 pandemic has emerged as an unprecedented healthcare crisis [1–3]. Increasing work demands on healthcare professionals cause psychological stress. Previous pandemics involving quarantine have emphasized that healthcare workers might develop symptoms of post-traumatic stress disorder, anxiety, depression, insomnia, and substance use disorders [4]. As the spread and burden of the pandemic varied by geographic regions (https://www.who.int/chp/about/regions/en), with overwhelming numbers of severe cases in some places [5], and only sporadic transmission with few in others suggesting that the psychological burden also varies across regions [6].

Preliminary reports from countries affected early on in the pandemic highlight the high prevalence of psychological burden in healthcare providers outside the critical care setting [7, 8]. First, in the general population exposed to the health crisis and state of emergency, high levels of loneliness, worry, fatigue, and low distress tolerance were reported during the early weeks of the COVID-19 pandemic. They were significantly associated with depression, anxiety, and stress symptoms [4, 9]. Second, this psychological burden appears to be higher in healthcare providers, especially in those who are younger, less experienced, and amongst those working in frontline [10]. Increased working hours, the number of Covid-19 patients cared for, and limited logistic support are associated with the highest prevalence of mental burden [10]. In a systematic review of 13 studies (33,062 participants), the pooled prevalence of anxiety and depression was 23.2%, and 22.8%, respectively [11]. In a study of healthcare providers exposed to COVID-19 patients, half of them self-perceived burnout [12]. In another study of 376 healthcare professionals in Italy, more than 1 out of 3 showed a high score of emotional exhaustion, and 1 out of 4 reported high levels of depersonalization, while around 15% reported low levels of personal accomplishment [13]. However, not all these studies captured the three major burnout domains, namely, emotional exhaustion, depersonalization, and personal accomplishment [14, 15], using valid instruments.

It is well recognized that the COVID-19 pandemic is putting healthcare professionals working in critical care under extreme pressure [7, 16]. Aside from the disruption of healthcare delivery in highly affected regions, the scarcity of resources such as personal protective equipment, ICU beds, and ventilators, increase this psychological burden [17]. Although features such as exhaustion, psychological disturbances, and stigmatization are to be expected, they are seldom documented [18]. Notably, the prevalence of burnout using a validated screening tool has never been reported in healthcare professionals working in critical care. In this context, to document the prevalence of mental health outcomes in ICU specialists facing the COVID-19 outbreak, we performed an online survey on behalf of the European Society of Intensive Care Medicine (ESICM).

## Methods

This web-based survey endorsed by ESICM collected data between April 30 and May 25, 2020, using an online questionnaire sent through the ESICM members’ list (https://www.surveymonkey.com/r/F2FFC6S). Online consent was obtained from the participants.

We collected demographic variables and information regarding personal and professional experience in managing severe COVID-19 patients. The number of COVID-19 patients managed as defined as the number of patients for whom the responding physician was providing direct care (clinical examination, medical prescription or procedures during day or night shifts). We collected data on the mental health outcomes included symptoms of severe burnout, anxiety, and depression, using the Hospital Anxiety and Depression Scale (HADS) and Maslach Burnout Inventory (MBI). The HADS is a 14-item auto questionnaire that includes 7 items about symptoms of anxiety, and 7 items on depression [19]. MBI consists of a 22-item questionnaire on the three components of burnout: emotional exhaustion (9 items), depersonalization (5 items), and personal achievement (8 items). Participants were classified as having symptoms of anxiety or depression when the corresponding subscale was > 7 [20] and high levels of burnout when the MBI was > −9 [15]. Visual analog scales were used by respondents to rank their relationship with ICU physicians, nurses, administrators, the quality of the decision-making and the ethical climate. The ethical climate was defined as “individual perceptions of the organization that influences attitudes and behaviour and serves as a reference for employee behavior” [21].

As ESICM has members worldwide, we summarized the demographic characteristics and categorized the respondents from 85 countries, into 12 different regions, as previously described [22]. Continuous variables are described as median (interquartile range [IQR]) and are compared between groups using the non-parametric Wilcoxon rank-sum test. Categorical variables are expressed as frequency (percentages) and are compared between groups using Fisher’s exact test. Factors independently associated with mental health outcomes were assessed using Cox model. Conditional stepwise variable selection was performed with 0.2 as the critical *P*-value for entry into the model, and 0.1 as the *P*-value for removal. Interactions and correlations between the explanatory variables were carefully checked. Statistical analyses were performed with R statistical software, version 3.4.3 (available online at http://www.r-project.org/). A *P*-value < 0.05 was considered significant.

## Results

Among ESICM members, 5660 opened the e-mail advertising for the survey, 1132 (20%) responded, including 1001 complete answers for demographic questions (Table 1), 848 complete responses for the Hospital Anxiety and Depression Scale (HADS) and 846 complete responses for the Maslach Burnout Inventory (MBI).

**Table 1:** Characteristics of responding physicians

Numbers (%) or median (interquartile ranges)	Total, 1001 respondents
Age	45 (39–53)
Female gender	342 (34.2%)
Single	170 (17%)
Number of children	2 (0–2)
Religiosity (0 not at all—100 very religious)	26 (1–61)
Current smoker	97 (9.7%)
Sleeping pills intake	374 (37.4%)
Excessive alcohol intake (self-report)	121 (12.1%)
Live in a city > 1 million inhabitants	403 (40.3%)
Work in a university-affiliated hospital	551 (55.1%)
Number of ICU beds baseline/during the surge	20 (11–36)/35 (20–60)
Number of night shifts per month	5 (3–6)
Number of COVID-19 patients managed	30 (14–60)
Ratings (0 poor–10 excellent)	
Relationship with doctors	8 (7–9)
Relationship with nurses	9 (8–9)
Relationship with administrators	7 (5–8)
Relationship with referring physicians	8 (7–9)
Quality of the decision-making	8 (7–9)
Ethical climate	8 (7–9)
Mental health outcomes	
Hospital anxiety and depression scale, anxiety subscale *N* = 848 respondents	7 (4–9)
Presence of symptoms of anxiety	395 (46.6%)
Hospital anxiety and depression scale, depression subscale	4 (2–7)
Presence of symptoms of depression	256 (30.2%)
Maslach burnout inventory N = 846 respondents	−8 (−21 to 8)
Presence of severe burnout	439 (51.8%)
Emotional exhaustion sub score	18 (10–29)
Depersonalization sub score	8 (4–12)
Personal accomplishment sub score	35 (29–40)

Respondents (45 years [39–53], 34% female) were mostly from Middle Europe (25%), Southern Europe (23%), the United Kingdom (12%), South America (9%), Northern Europe (8.1%), Eastern Europe (5.3%), Middle-East (5%), North America (4.7%), Asia (3.3%), India (2.7%), Australia–New Zealand (1.3%) or Africa (0.6%); 54% were living in a city of > 1 million inhabitants, and 55% were working in a University-affiliated hospital. 37.2% intensivists took sleeping pills, 12% reported having excessive alcohol intake, and 9.7% were smoking.

**Fig. 1:**
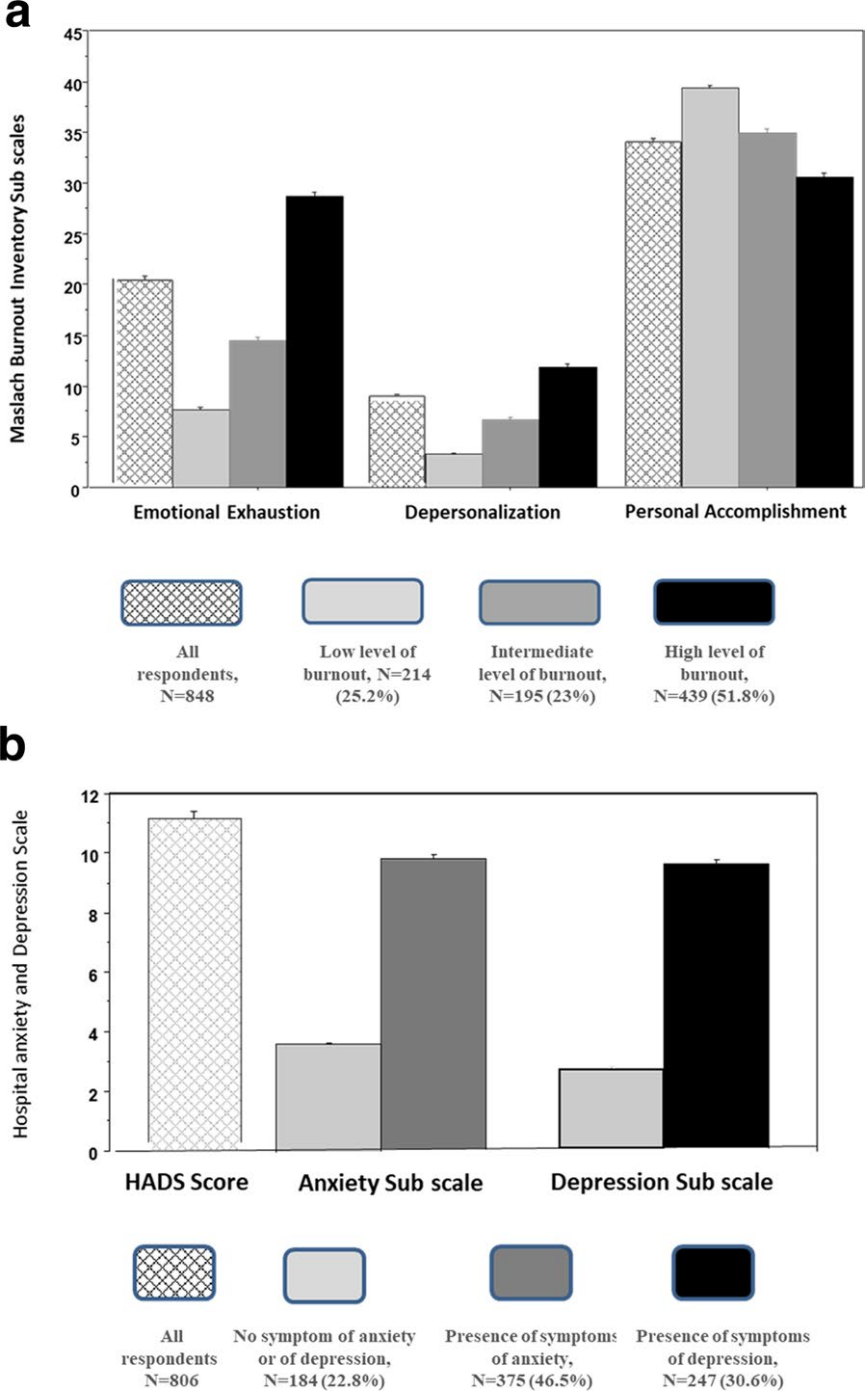
Maslach and Burnout Inventory in 846 ICU specialists. The three domains of the instrument, namely emotional exhaustion, depersonalization, and personal accomplishment, are displayed separately. Symptoms of emotional exhaustion are mild or severe in 29.9% and 23.0% of the respondents. Symptoms of depersonalization are mild or severe in 34.3% and 23.0% of the respondents. Symptoms of personal accomplishment are mild or severe in 35.2% and 31.4% of the respondents. Overall, prevalence of severe burnout was reported in 51.8% (439/846) of the respondents

**Fig. 2:**
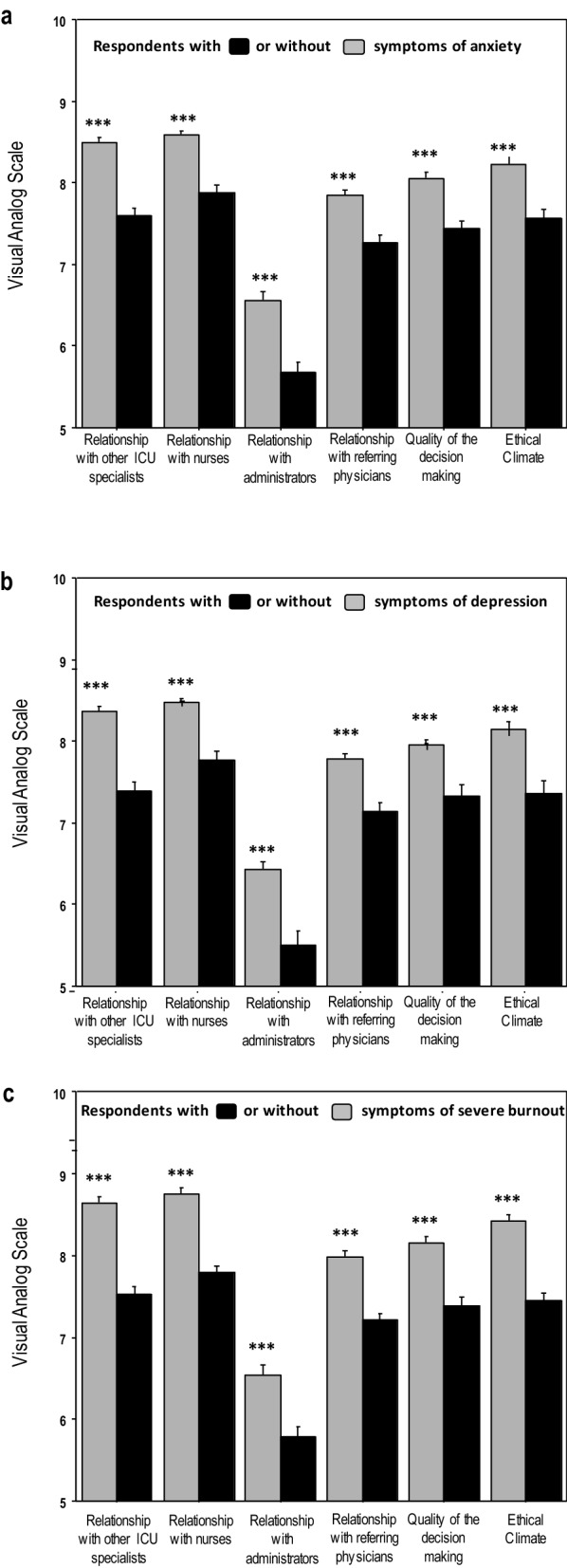
Physician’s ranking (from 0 [very poor relationship] to 10 [excellent relationship]) of their relationship with other ICU specialists, nurses, administrators, or with referring physicians. They have also ranked the quality of the decision-making and the ethical climate in their ICU. Results are presented according to the presence of symptoms of anxiety (**a**), the presence of symptoms of depression (**b**), or the presence of symptoms of severe burnout (**c**). This figure displays the results in the 848 ICU specialists returning complete HADS. *** indicates *P* value < 0.0001

Regarding the mental health outcomes of ICU specialists during the COVID-19 outbreak, the prevalence of symptoms of anxiety, depression, and severe burnout were 46.5%, 30.2%, and 51%, respectively (Fig. 1). Ranking (VAS from 0 [poor] to 10 [excellent]) of the relationship between ICU specialists and other ICU specialists, nurses, administrators, or primary physicians were significantly different according to the presence of symptoms of anxiety (Fig. 2, panel a), depression (Fig. 2; panel b) or severe burnout (Fig. 2, panel c). Similarly, physicians with either of these symptoms provided significantly lower scores to the ethical climate or the quality of the decision-making ratings (Fig. 2).

**Fig. 3:**
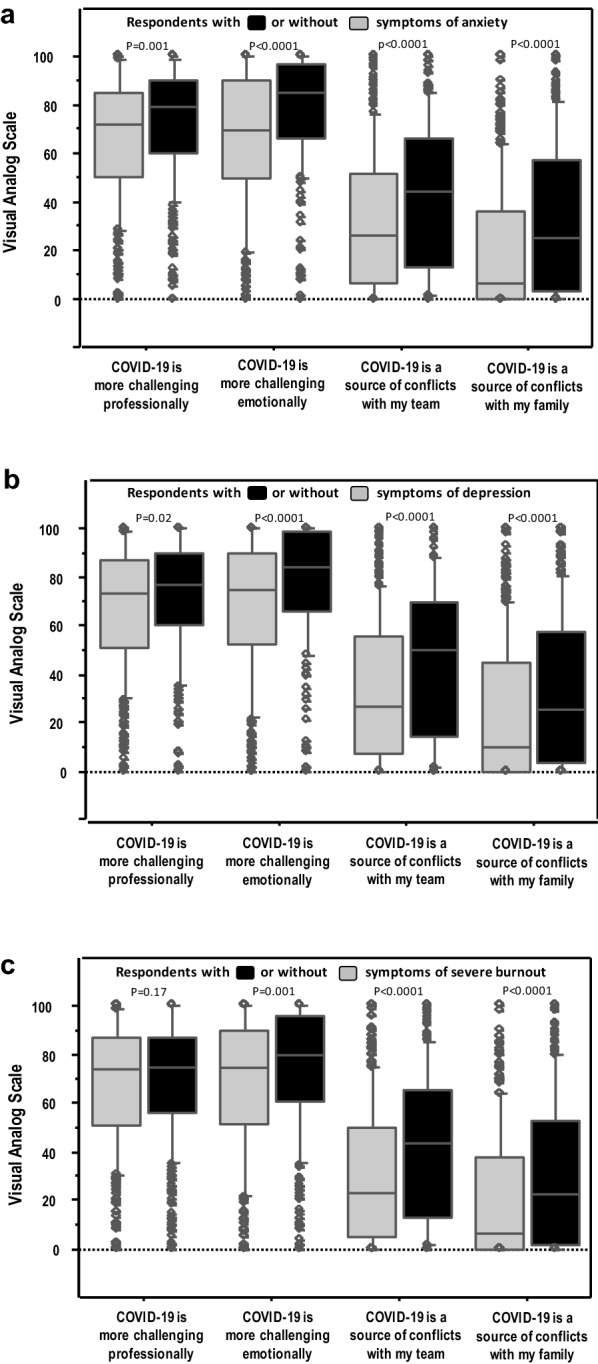
Physician’s ranking (from 0 [less challenging than non-COVID patients] to 100 [more challenging than non-COVID patients]) of how the pandemic has been a professional and emotional challenge, and whether it was a source of intra-team or intra-family conflict. Results are presented according to the presence of symptoms of anxiety (**a**), the presence of symptoms of depression (**b**), or the presence of symptoms of severe burnout (**c**)

Respondents were asked to compare their COVID-19 experience to general ICU non-COVID patients in a 0 (less challenging than non-COVID patients) to 100 (more challenging than non-COVID patients) scale. Overall, the scores reported were (75 [55–88]) for the professional challenge, (80 [59–93]) for the emotional challenge, (32 [8–61]) for the intra-team conflicts, and (12 [0–50]) for the intra-family conflicts. To the question of whether this experience would make them see their professional career differently, ICU specialists provided a score of 50 (4–75). Figure 3 shows the distribution of the scores in ICU specialists with and without symptoms of anxiety (panel a), depression (panel b), or severe burnout (panel c).

By univariable analysis, factors associated with symptoms of anxiety were female gender (41.2% in those with symptoms of depression vs. 27.9% in those without), younger age (44.5 [38–52] vs. 47 [40–54], years *P* = 0.0002), living in city of > 1 million inhabitants (50.2% vs. 42.1%, *P* = 0.02), and higher religiosity (35 [4–66] vs. 19 [0–57], *P* = 0.001). Factors associated with the prevalence of symptoms of depression were female gender (42.4% in those with symptoms of depression vs. 30.5% in those without, *P* = 0.0008), younger age ((44 [38–51] vs. 46 [39–54], *P* = 0.004), being single (21.4% vs. 15.1%, *P* = 0.03), living in city of > 1 million inhabitants (63.6% vs. 49.5%, *P* = 0.0003), and higher religiosity (36 [5–67] vs. 21 [0–59], P = 0.002). Working in a university-affiliated hospital was associated with a lower prevalence of symptoms of anxiety (42% vs. 52%, *P* = 0.007) but did not affect the prevalence of symptoms of depression. Age and female gender were also associated with a higher prevalence of severe burnout (45 [37–51] vs. 47 years [40–55], *P* = 0.0001, and 38.2% vs. 30.1%, *P* = 0.02). Clinicians with symptoms of anxiety, depression, or severe burnout were more frequently smoking or taking sleeping pills, whereas alcohol consumption was not affected. The number of COVID-19 patients managed was not associated with the prevalence of the psychological burden.

**Fig. 4:**
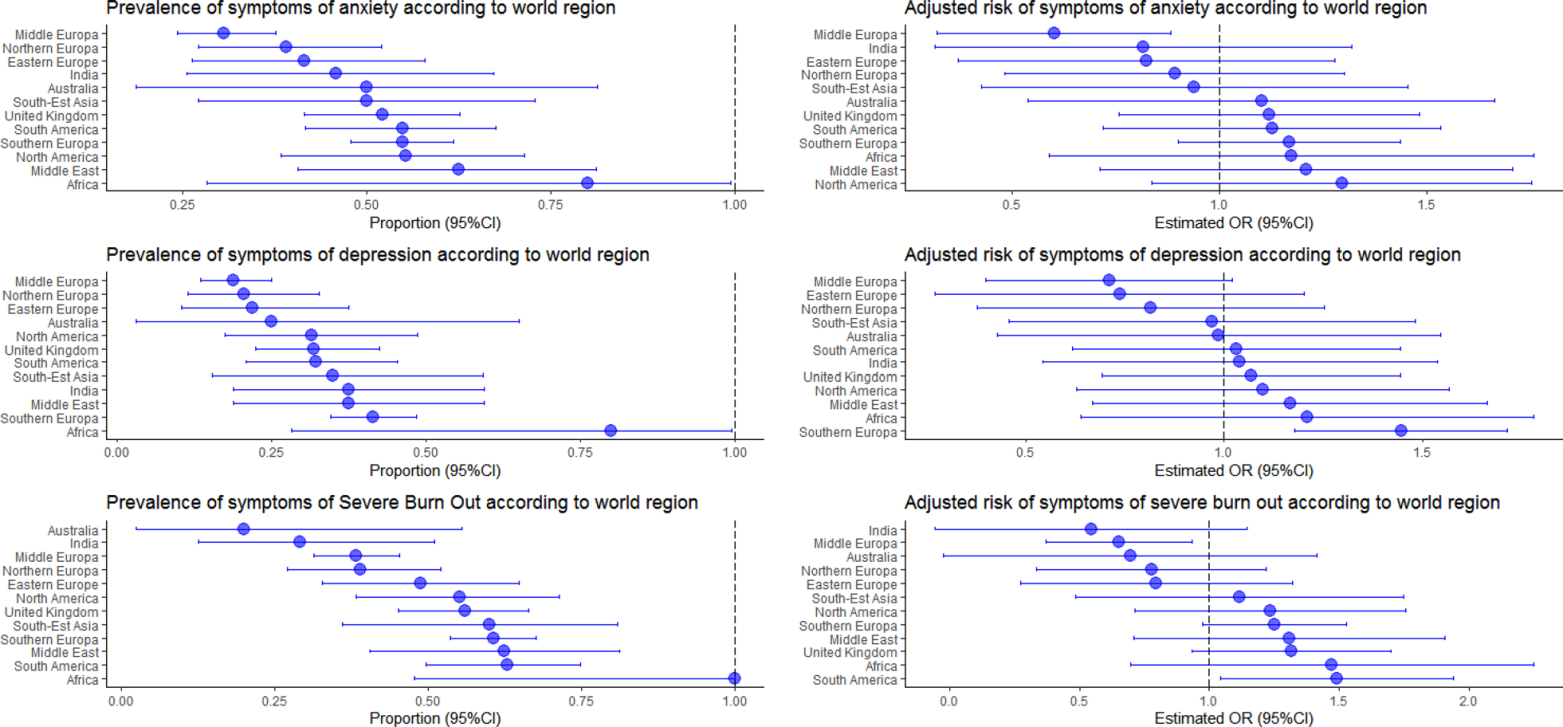
Prevalence and adjusted prevalence of anxiety, depression and severe burnout in the 12 participating regions

**Fig. 5:**
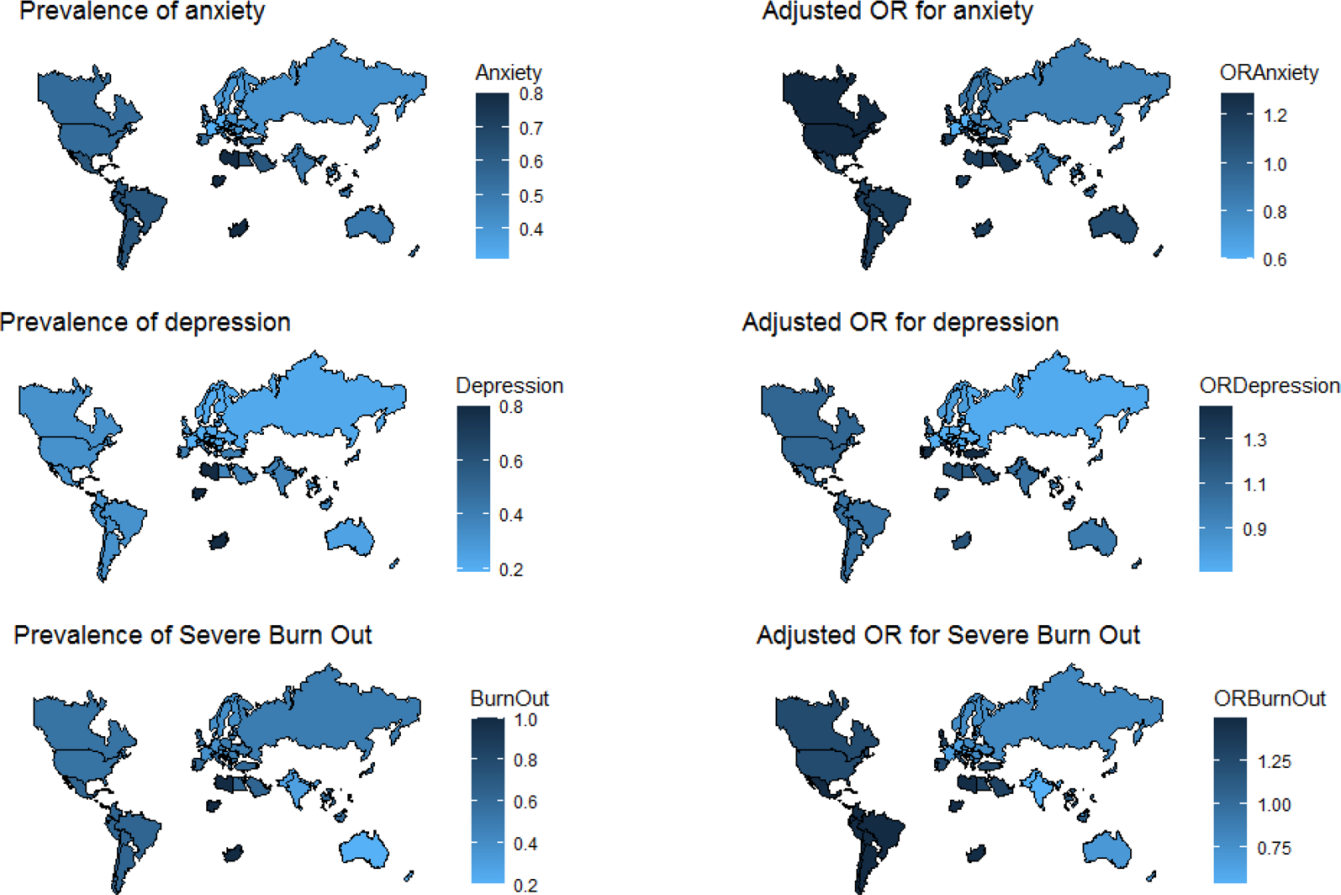
World map displaying the prevalence (graphs in the left) and the adjusted odds ratio (OR, graphs in the right) of symptom of anxiety, depression and severe burnout across regions

The prevalence of symptoms of anxiety, depression, and burnout varied significantly across regions (Figs. 4 and 5). For anxiety, Middle Europe, East Europe, Asia, and Scandinavia were in the 30–40% range, Australia–New Zealand, South America, North America, the UK, and South Europe in the 50–60%, and India, Middle-East and Africa in the > 60% range. For depression, Middle Europe, Australia–New Zealand, Scandinavia, and East Europe were in the 20–40% range, the UK, North America, Asia, South America South Europe, and the Middle East in the 30–50%, and Africa was 80%. For severe burnout, Australia–New Zealand, India, Middle Europe, and Scandinavia were in the 20–40% range, East Europe, North America, Asia, South America, the UK, South Europe, and the Middle East were in the 50–70% range.

By multivariable analysis, factors independently associated with symptoms of anxiety were female gender (HR 1.85 [1.33–2.55]), working in a university-affiliated hospital (HR 0.58 [0.42–0.80]), living in a city of > 1 million inhabitants (HR 1.40 [1.01–1.94]), and clinician’s rating about the ethical climate (HR 0.83 [0.77–0.90]). Factors independently associated with symptoms of depression were female gender (HR 1.63 [1.15–2.31]) and clinician’s rating about the ethical climate (HR 0.84 [0.78–0.92]). Factors independently associated with symptoms of severe burnout included age (HR 0.98/year [0.97–0.99]) and clinician’s rating about the ethical climate (HR 0.76 [0.69–0.82]).

## Discussion

The novel SARS-CoV-2 pandemic has resulted in an overall surge in new cases of anxiety, depression and burnout in critical care health care workers. Determinants of mental health outcomes included clinician’s characteristics (age, gender, religiosity), ICU characteristics (region, located in a large city, university-affiliated), and how critical care specialists ranked the ethical climate in their ICU.

Given the highly contagious nature of SARS-CoV-2, occupational hazards have been associated with more emotional impact in physicians who work on the frontline [10]. However, this study, including ICU specialists managing critically ill COVID-19 patients, did not confirm that exposure to the disease or the physical strain associated with the surge were the leading factors for symptoms of severe burnout, anxiety, and depression [10, 12, 13]. The number of COVID-19 patients managed, the number of night shifts per month, or time since the last vacation at the time of the surge were not associated with either symptom.

The substantial variation in the prevalence of burnout across regions calls for attention [23]. Indeed, the pandemic exposed ICU specialists to the inadequacy of national stockpiles in personal protective equipment, ventilators, staff, and drugs [24–26]. National responses, including lockdown timing and duration, have varied also worsening the psychological pressure on the personnel. Moreover, the lack of established policies for patient’s triage and management has increased the burden on health care workers [27]. Each professional society has issued guidelines potentially mismatching with local procedures or with recommendations from other specialties, with no effort to align such guidelines. Last, as the pandemic occurred heterogeneously and asynchronously throughout the world, both the COVID-19 burden and the effects of the lockdown were captured at different time points from the disease peak [4].

Even though anxiety and depression have not been assessed using the HADS, this study reports a prevalence of burnout in ICU physicians of 52%. These results suggest then that the SARS-CoV-2 pandemic has generated more burnout than what could have been expected. For instance, in the systematic review from van Mol et al. [28], reported rates of burnout in ICU physicians were about 30% when the MBI was used [29–32].

Moral distress from suboptimal decision-making, difficulties in involving the relatives, and the perception of inappropriate care may be a cornerstone for the development of psychological burden in ICU specialists. Moral distress is an ethical root cause of clinician burnout [21], translating in low clinician well-being, job dissatisfaction, and job turnover [33]. During the pandemic, tough decisions for patients lacking decision-making capacity had to be made without any relative [34]. Clinicians struggled to balance substituted judgment with their views on the best interests of the patient, creating a climate for suboptimal decision-making [35]. In this study, the quality of the decision-making was associated with the prevalence of symptoms of anxiety, depression, and burnout. Ethical climate should also be recognized as an essential contributor that either alleviates or exacerbates moral distress [33]. Here again, the ranking of ethical climate was an independent predictor of anxiety, depression, and severe burnout. However, studies are needed to ascertain whether the ethical climate could be a surrogate of optimal teamwork in the setting of the COVID-19 pandemic where many difficult decisions were also made in the ED or the wards.

This study has several limitations. First, as an online study with a 20% response rate, these results might appear as biased. However, burnout syndrome has not been assessed in the setting of the COVID-19 pandemic. Moreover, with 1001 respondents from 85 countries and 12 regions, this is among the most extensive studies assessing severe burnout in ICU healthcare workers and intensivists. Second, the survey focused on frontline clinicians managing the COVID-19 pandemic, with no control group of COVID-19 naïve physicians. As the study was embedded in a COVID-19 survey, clinicians working in places not involved in the pandemic have considered themselves as non-concerned by the subject. However, the lack of association between the number of COVID-19 patients managed and the psychological burden suggests that other non-professional factors contributed to the mental alterations. Third, as half the respondents were from university-affiliated hospitals, a selection bias should be considered. Fourth, the data could not be captured when all of the participating countries were at the peak of the pandemic. Psychological symptoms may vary over time and this could explain part of the regional variability. Last, the restriction of this study to ICU physicians only provides only a partial picture of what post-COVID-19 psychological burden is. Studies assessing mental health outcomes in relation with the pandemic and including all healthcare professionals are warranted.

In summary, the pandemic has had an overwhelming impact on ICU specialists. With about half the ICU physicians having symptoms of severe burnout and anxiety as well as 30% with symptoms of depression, we can consider that COVID-19 has generated a mental health emergency. The lack of knowledge and experience about the disease was most probably a source of anxiety. Clinicians ranked this burden as the most significant professional and emotional challenge when compared to non-COVID-19 patients. These mental health problems may be generated by a failure to triage appropriately, manage and make decisions for the patients and the lack of an optimal ethical climate, more than the exposure to the disease or the physician strain form the surge. Physicians follow-up, and management are warranted to assess long-term psychological outcomes of the COVID-19 outbreak and alleviate the psychological burden of the pandemic on exhausted professionals.

## Data Availability

Fully available upon request.
